# Ultrasound-guided muscle biopsy: a practical alternative for investigation of myopathy

**DOI:** 10.1007/s00256-020-03484-y

**Published:** 2020-06-09

**Authors:** Anish Raithatha, Mohammad Reza Ashraghi, Christopher Lord, Clara Limback-Stanic, Stuart Viegas, Dimitri Amiras

**Affiliations:** 1 Department of Imaging, Imperial College Healthcare NHS Trust, London, UK; 2 Neuromuscular Unit, Department of Neurology, Imperial College Healthcare NHS Trust, London, UK; 3 Department of Cellular Pathology, Imperial College Healthcare NHS Trust, London, UK

**Keywords:** Myopathy, Muscle, Biopsy, Ultrasound

## Abstract

**Objective:**

We propose the use of ultrasound-guided muscle biopsy as a viable method of obtaining muscle specimen to aid the diagnosis of myopathy. We retrospectively review the diagnostic accuracy and patient feedback of ultrasound-guided muscle biopsies in our neuromuscular service.

**Method:**

Multidisciplinary team meeting reviewed select patients and agreed on those suitable for ultrasound-guided muscle biopsy. They then underwent biopsy using direct ultrasound guidance and a modified Bergström needle. The specimens were sent for histopathological analysis, and patients were given a feedback form.

**Results:**

Ten patients underwent 11 ultrasound-guided muscle biopsies. Of these 11, one was processed incorrectly, but all others were good quality specimens suitable for analysis. All 10 of those processed correctly aided diagnosis. All patient feedback was rated good or excellent. In 4 patients with a previous unsuccessful surgical biopsy, ultrasound-guided biopsy was successful in obtaining suitable muscle. Of those 4 patients, 3 preferred ultrasound-guided biopsy, and 1 did not state a preference.

**Discussion:**

Our ultrasound-guided muscle biopsy technique offers a viable alternative to surgical biopsy. It yields high-quality specimen that aids diagnosis and receives good feedback from patients. It can be performed quickly as a day case and does not require theatre space. Furthermore, direct visualization of structures minimizes the risk of complications and allows biopsy of otherwise difficult to access targets.

**Conclusion:**

Utilization of ultrasound guided–modified Bergström needle technique for muscle biopsy provides comparable success rates to other techniques and has practical, clinical, operational, and patient-centred benefits compared with alternative techniques.

## Introduction

Muscle biopsy is an essential diagnostic procedure in the investigation of the diagnosis of myopathy [1]. Muscle biopsies to aid the diagnosis of neuromuscular pathology require careful preservation of cellular architecture. In the UK, both percutaneous needle and open surgical biopsy are utilized. Both can be done as day-case procedures under local anaesthetic unless there are mitigating circumstances. Each has its own advantages and disadvantages [2]. The latter are the preferred choice in our institute as they provide a greater tissue yield, which is particularly important in suspected genetic cases. This requires both neurosurgical expertise and theatre space. In some cases, surgical biopsies have not been possible, and exploration of alternative methodologies to obtain adequate muscle biopsy samples is therefore of benefit to patients and their care.

**Fig. 1:**
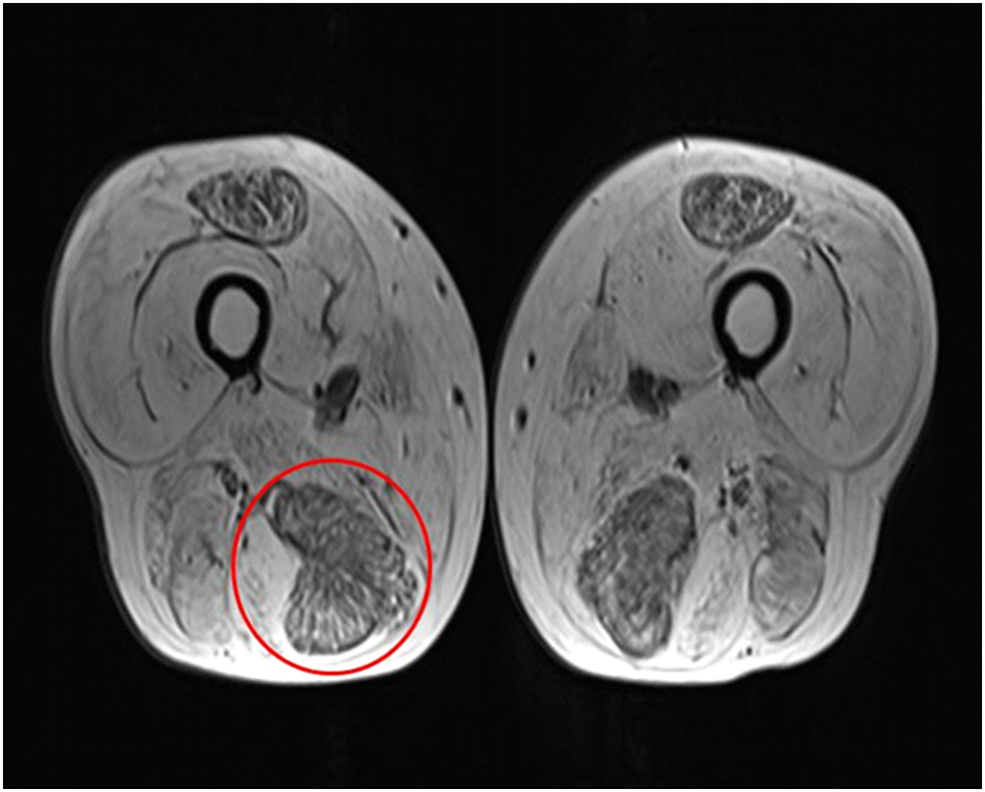
Axial T1 MRI image of the thighs, demonstrating fatty atrophy of the majority of the muscle groups and relatively sparing of the rectus femoris and semimembranosus muscles bilaterally. The right semimembranosus was selected for biopsy (circled), based on its reduced fat content

Local protocol utilizes a combination of clinical evaluation and magnetic resonance imaging (MRI) routinely to identify suitable targets for muscle biopsy by open surgical biopsy procedure. Axial T1-weighted and short T1 inversion recovery (STIR) sequences with high bandwidth are used to identify muscles that do not demonstrate significant fat content with preference given to those demonstrating features of active disease with intrinsic muscle and peri-fascial oedema (Fig. 1).

Despite this pre-procedural planning, muscle biopsies can be non-diagnostic. This can be due to a combination of the limited accuracy of intraoperative identification of viable muscle, varied distribution of disease within a muscle group and the constraint of access to target muscles [2]. A retrospective review of 106 patients undergoing muscle biopsy demonstrated a diagnostic pathologic yield of 47% [3].

To complement the surgical technique, we have initiated an ultrasound-guided muscle biopsy service utilizing a modified Bergström needle and vacuum technique. This allows the targeting of muscles previously not suitable for a surgical biopsy. In addition, this allowed targeting of areas of viable muscle within a single muscle group that were previously non-diagnostic surgically.

## Aims and objectives

The aim of this study is to retrospectively review the diagnostic accuracy and patient feedback outcomes of our ultrasound-guided muscle biopsy service that is utilized for the investigation of myopathy.

## Materials and methods

At our muscle multidisciplinary team (MDT) meeting, selected difficult cases of patients with undiagnosed myopathy, where an open surgical biopsy had been non-diagnostic or a surgical biopsy was deemed too technically challenging, were discussed. The challenges to more traditional surgical biopsy included the need for a larger incision to confirm anatomy of smaller target muscles and the risk of damage to surrounding neurovascular structures. Furthermore, it was felt that obtaining viable muscle by surgical biopsy in some patients with extensive muscle disease would be challenging. In these patients, US guidance was considered to help identify viable muscle and therefore improve diagnostic yield. If it was felt to be feasible by the musculoskeletal radiologist, a target muscle was identified on MRI using the same T1 and STIR sequences used for planning surgical biopsies, and an ultrasound-guided muscle biopsy was scheduled. More commonly performed deltoid and quadricep biopsies were still routinely sampled by open surgical biopsy.

**Fig. 2:**
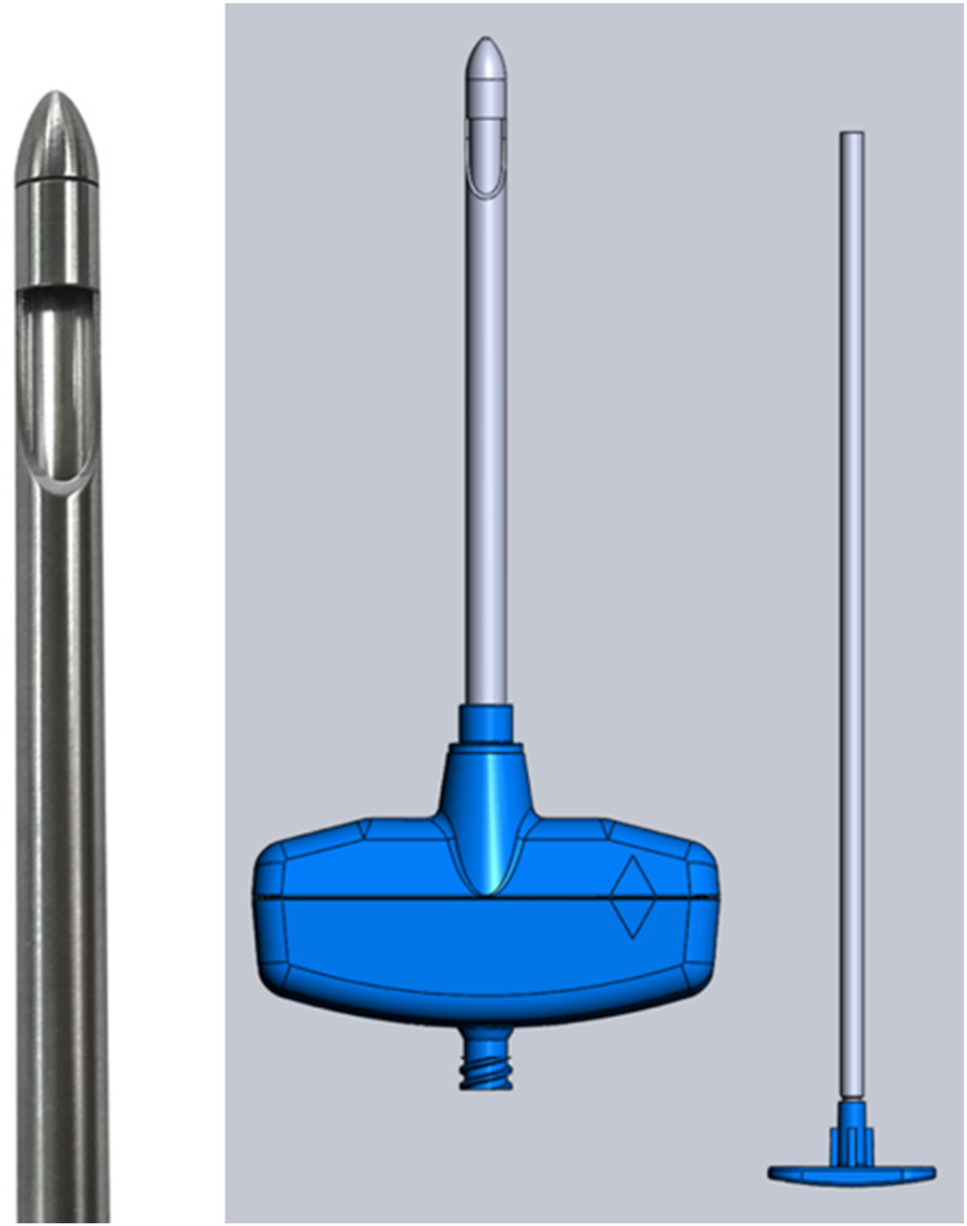
Modified Bergström needle demonstrating plastic handles and the blunt tip [5]

**Fig. 3:**
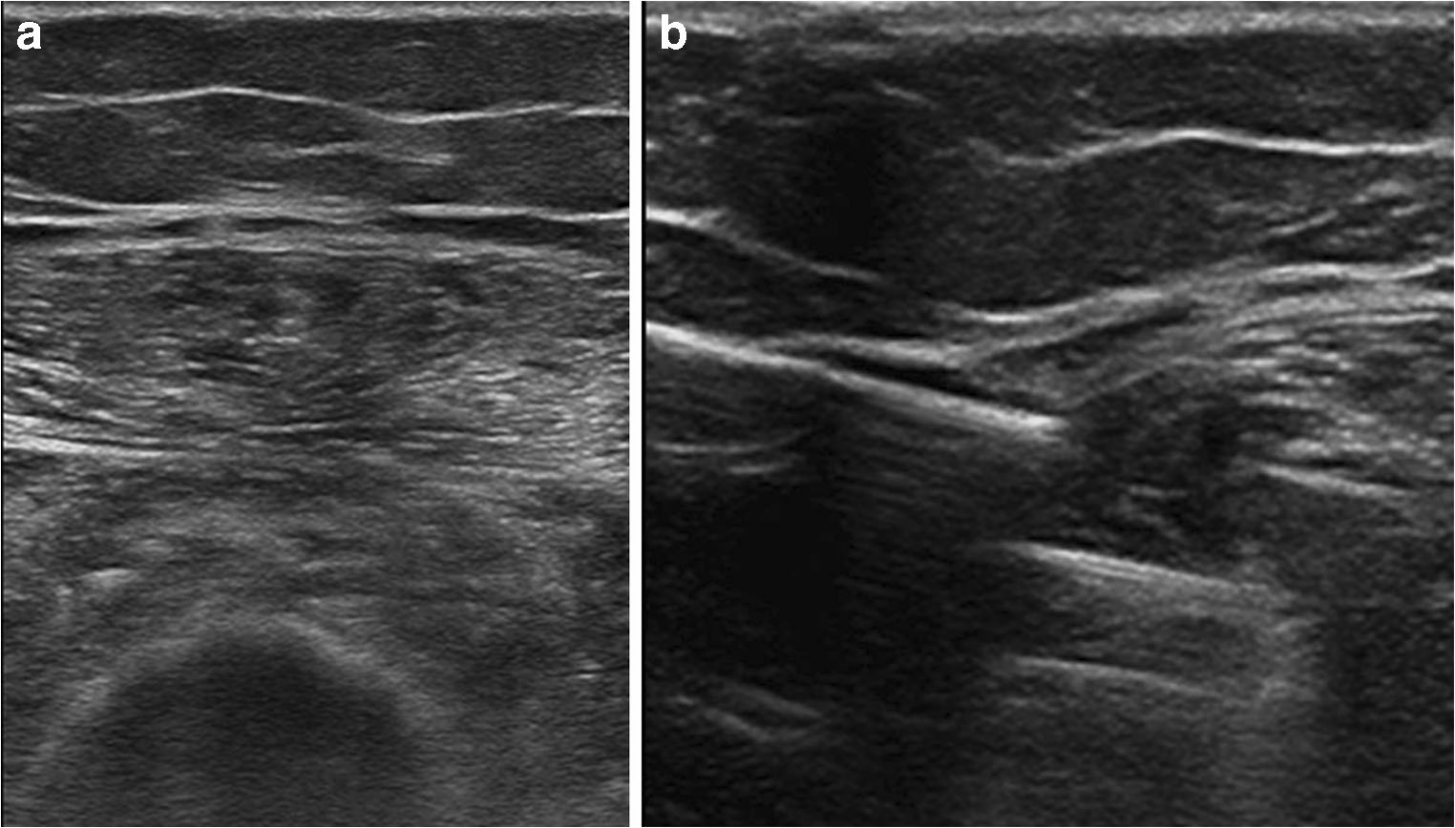
**a** Ultrasound of the leg demonstrates an expanded, hyperechoic semimembranosus muscle. **b** The muscle was targeted under real-time ultrasound guidance and biopsy samples retrieved utilizing a modified Bergström needle and suction technique. The image shows the gap for the biopsy and identifies the muscle fibres of the semimembranosus muscle being sampled

A modified Bergström needle with a 5 mm core diameter was used. This was altered from the original Bergström design by the manufacturer to be disposable with plastic handles and a blunt tip (Fig. 2) [4, 5]. A vacuum technique was performed under ultrasound guidance to better target a previously identified muscle (Fig. 3a).

Ultrasound with an appropriate transducer for the depth was used to identify the target muscle, and in most cases, this was 12–15 MHz. Local anaesthetic was infiltrated into the subcutaneous tissue and fascial plane with a 21G needle and into the peri-muscle facia with a 21G spinal needle. Up to 10 ml of 1% lidocaine was used with 10 ml normal saline to achieve good local anaesthesia. If possible, the posterior muscle wall fascia was also targeted; however, great care was taken not to infiltrate the muscle itself. Fascial perforation was then performed using a scalpel under direct sonographic guidance. The scalpel was identified under ultrasound and advanced to the facia where a small longitudinal incision was made in the muscle fascia. This avoided any significant vascular structures and facilitated the passage of the blunt tipped modified Bergström needle into the muscle compartment under a small amount of pressure. The Luer lock at the end of the biopsy needle was then attached to wall suction at a maximum pressure of 500 mmHg, and the modified Bergström needle was then advanced to the target muscle under continuous sonographic guidance (Fig. 3b).

Once the needle was passed into the muscle, the stylet was advanced, and up to 3 passes were performed to obtain at least 2 samples. Each sample was directly visually inspected for muscle tissue and placed in a universal container and sent for immediate histopathological analysis. The biopsy sample was approximately 5–10 mm in length.

After the procedure, each patient was observed while asked to lie on the site of biopsy for 2 h. Once stable, the patient was discharged.

Post-biopsy, patients were reviewed on an outpatient basis, determined by clinical need.

Histology reports for the ultrasound-guided procedures were reviewed to confirm if this technique had allowed adequate diagnosis.

In addition, each patient undergoing the procedure was retrospectively surveyed and asked to comment on any scar, complication and if they would favour the procedure in the future. Those that had had a prior surgical biopsy were asked to state a preference too.

## Results

**Fig. 4:**
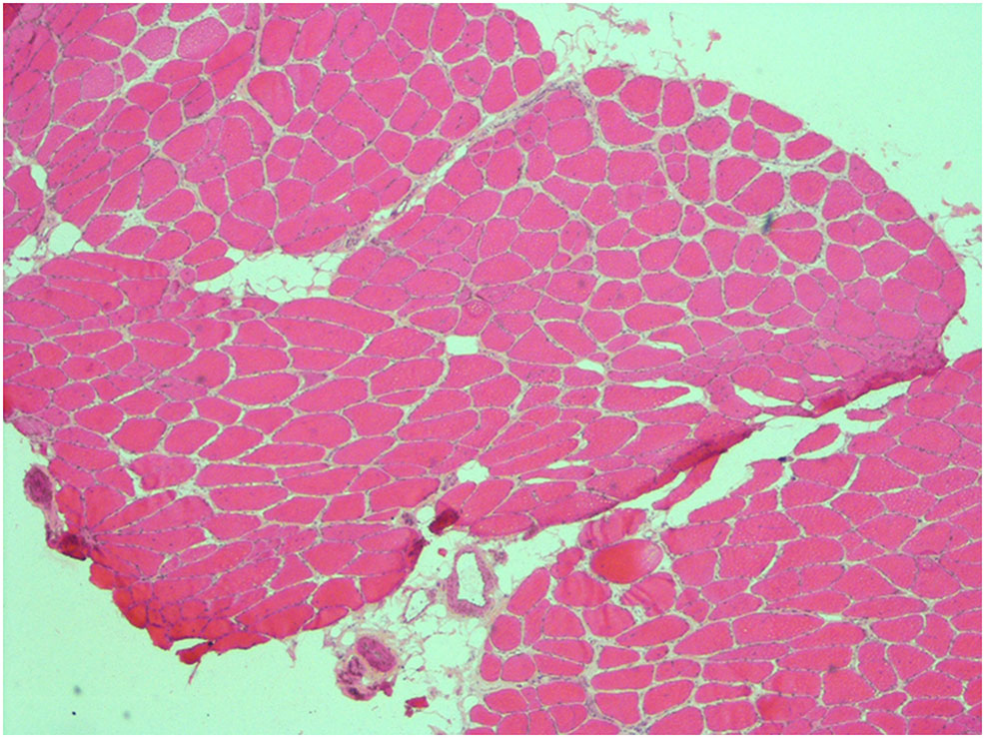
Frozen section, HE staining: a representative section at low-power magnification shows good tissue quality and proper orientation of the specimen

Over a 2-year period, a cohort of 10 patients met criteria to undergo the ultrasound-guided muscle biopsy using a modified Bergström needle and suction technique. These patients underwent a total of 11 biopsies during this time. Table 1 summarizes the outcome of these. The biopsies were reviewed by our neuropathologists who were satisfied with the quality and quantity of the tissue and were able to carry out the necessary stains to aid a diagnosis. This is well demonstrated by the histology sample shown below (Fig. 4).

**Table 1:** Summary of ultrasound-guided muscle biopsy outcome and feedback

Patient #	Previous surgical biopsy	Complications	Appropriate sample	Change in pathological diagnosis	Diagnosis	Feedback available	Patient experience **	Preferred to surgical biopsy (if applicable)
1	Yes	No	Yes	Yes	Systemic vasculitis	Yes	5	Yes
2	No	No	Yes	Yes	Jo-1/Ro52 associated inflammatory myopathy	Yes	4	N/A
3	Yes	No	Yes	Yes	Autophagic vacuolar myopathy	Yes	4	No response
4	No	No	Unknown*	No	Myopathy	Yes	4	N/A
4	No	No	Yes	Yes	Possible LGMD	No	N/A	N/A
5	No	No	Yes	Yes	Normal muscle	No	N/A	N/A
6	No	No	Yes	Yes	Axial myopathy	No	N/A	N/A
7	Yes	No	Yes	Yes	Sporadic late-onset nemaline rod myopathy	Yes	5	Yes
8	Yes	No	Yes	Yes	Anti-SRP + ve necrotising myopathy	Yes	5	Yes
9	No	No	Yes	Yes	Likely IBM	Yes	5	N/A
10	No	No	Yes	Yes	Axial myopathy	No	N/A	N/A

*Biopsy transported in formaldehyde

**0 (extremely poor), 5 (excellent)

A retrospective review demonstrated that ten of the eleven samples were of satisfactory diagnostic quality and permitted histological analysis. The one sample that was non-diagnostic in quality was inappropriately transported in formaldehyde following the biopsy. The biopsy for this patient was successfully repeated. One patient had vastus lateralis and a paraspinal muscle biopsied. For this patient, the vastus lateralis was diagnostic, but the paraspinal was not. Furthermore, all ten of the successful ultrasound-guided biopsies aided a diagnosis, either by confirming non-specific muscle pathology, by directly supporting a specific diagnosis or by excluding a specific diagnosis.

Patient feedback from the procedure was encouraging, and no significant complications were reported. Four patients had undergone a surgical biopsy previously. Three of these preferred the ultrasound-guided biopsy to the surgical approach, while one did not specify. No complications were reported from the eleven biopsies. When asked to rate their experience between 0 and 5, with 0 being extremely poor and 5 being excellent, eight patients rated 4 or 5. The others did not fill this part of the questionnaire.

Furthermore, of the four patients who had had a previous surgical biopsy that had been unsuccessful in establishing a diagnosis, an ultrasound-guided biopsy was successful in aiding a diagnosis in all. This was particularly well demonstrated in a case of a 55-year-old patient with a history of myopathic weakness since his teenage years affecting all limbs and extraocular muscles. The MRI of his muscles had shown marked atrophy and fatty infiltration (Fig. 1), making a viable muscle biopsy target difficult to select. This was discussed with the neurosurgical team who did not feel they could reliably biopsy muscle. He had already undergone two surgical muscle biopsies over the preceding decade which had been non-diagnostic. Therefore, after discussion in muscle MDT, he underwent a successful ultrasound-guided muscle biopsy using the aforementioned technique. This high-quality biopsy (Fig. 4) has aided us in diagnosing an autophagic vacuolar myopathy that is suspected to be a new case of X-linked myopathy with excessive autophagy (XMEA).

## Discussion

Image-guided muscle biopsy is performed at multiple institutions globally, utilizing either MRI or sonographic guidance [6, 7]. However, current evidence demonstrates that sonographic guidance is associated with fewer bleeding complications and is more cost-effective than utilization of MRI due to the longer procedure time and use of disposable materials [7]. Current studies have not utilized a modified Bergström needle technique in combination with ultrasound guidance, and therefore our data set is the first to evaluate the use of this approach with ultrasound.

Ultrasound-guided percutaneous muscle biopsy has been demonstrated to provide diagnostic rates comparable with open biopsy in the assessment of acute muscle disease but less successfully for chronic disease [8]. Furthermore, this technique conveys further practical benefits: the modified Bergström needle and suction technique is best utilized for difficult to access muscle groups that cannot be targeted using an open approach as it requires a smaller incision and reduces use of surgical theatre time. The procedure is also rapid and can be performed in an outpatient setting under local anaesthesia. Visualization of key structures minimizes the risk of post procedural bleeding and other complications, while also aiding the identification of viable muscle for biopsy. Additionally, the procedure is better tolerated by patients in comparison with surgical methods.

Our current data demonstrates a 100% success rate for utilization of an ultrasound-guided approach to gaining appropriate specimens in cases where open surgical biopsy has been inconclusive and thereby establishes the use of US-guided biopsy as a viable practice to facilitate accurate histological diagnosis in this patient subcohort. The potential benefit of this approach is particularly demonstrated in the case described above. Furthermore, we were able to obtain at least one suitable muscle biopsy that was diagnostically helpful in 100% of our patients using ultrasound guidance.

The procedure was well tolerated by patients with minimal bleeding. No complications were reported by patients. At least half of those who have previously had a surgical biopsy expressed a preference for the ultrasound-guided approach, and none expressed a preference for surgical biopsies.

## Conclusion

In the context of establishing a neuromuscular diagnosis, we demonstrated benefit in utilizing an ultrasound guided–modified Bergström needle technique to obtain viable histological samples in patients that have had an unsuccessful open biopsy procedure.

Previous studies have utilized this technique with MR guidance, but our data shows that an ultrasound-guided procedure infer additional advantages, providing comparable success rates to other techniques, and have practical, clinical, operational and patient-centred benefits compared with alternative techniques.

Furthermore, the procedure was not associated with significant complication and was well tolerated by patients with positive feedback.
